# Editorial: Engineering Synthetic Metabolons: From Metabolic Modeling to Rational Design of Biosynthetic Devices

**DOI:** 10.3389/fbioe.2016.00039

**Published:** 2016-05-06

**Authors:** Lars M. Voll, Zoran Nikoloski

**Affiliations:** ^1^Division of Biochemistry, Friedrich-Alexander-Universität Erlangen-Nürnberg, Erlangen, Germany; ^2^Max-Planck-Institute for Molecular Plant Physiology, Potsdam-Golm, Germany

**Keywords:** synthetic biology, pathway engineering, bioreactors, model-based design, computational models, molecular dynamics modeling

**The Editorial on the Research Topic**

**Engineering synthetic metabolons: from metabolic modelling to rational design of biosynthetic devices**

Synthetic Biology is an emerging discipline that enjoys rapidly increasing popularity. However, there is no widely accepted definition for the term *Synthetic Biology*, because the field of Synthetic Biology is multifaceted and rapidly moving. As viewed from its underlying principle, Synthetic Biology can be defined as the “application of engineering principles to biological systems.” It reaches from pathway engineering in living systems, i.e., the introduction of functional biochemical pathways into existing organisms (see featured research topic review by Pröschel et al. and references cited therein), and the design of vesicle-based multicompartmented biochemical reactors and protocells (see featured research topic review by Schmitt et al. and references cited therein) to the creation of entirely synthetic cells with reproductive potential that is encoded by synthetic genes [with the work of Gibson et al. (2008, 2010) that attracted the greatest publicity in the recent years]. Likewise, the definition provided at http://syntheticbiology.org covers these two extremes in that Synthetic Biology is defined as “(A) the design and construction of new biological parts, devices, and systems and (B) the re-design of existing, natural biological systems for useful purposes.” This also means that Synthetic Biology is not limited to living systems but also comprises the combination and re-design of biological parts into artificial bioreactors. In this respect, Synthetic Biology is highly interdisciplinary, bringing together molecular biology with biophysics, material sciences, bioengineering, and computational approaches.

Besides the invaluable role in helping biochemists understand the metabolic complexity of living systems and the extent to which observations from existing metabolic engineering strategies match predictions from large-scale modeling (see, e.g., the featured research topic article by Basler et al.), models are crucial for the rational design of synthetic systems (see, e.g., the featured research topic article by Elbinger et al.). Metabolic modeling has the capacity to guide the biochemical layout of engineered pathways *in vivo* and the design of artificial bioreactors *in vitro*. For instance, computational modeling can play an important role for the selection of individual enzyme isoforms for engineered metabolic pathways. *Vice versa*, *in vitro* studies in artificial bioreactors has advanced our understanding of biochemically complex processes, such as starch biosynthesis, as summarized in the featured research topic review by O’Neill and Field. On the other hand, molecular dynamics simulations help to understand complex biochemical and biophysical processes such as enzymatic catalysis, molecular interactions of biomolecules (see featured research topic reviews by Horn and Sticht and Groß et al.), or membrane transport (see featured research topic articles by Róg et al., Bertelshofer et al. and Sun et al.). The knowledge gained by these simulations can be exploited for the structural and molecular design of synthetic systems, i.e., for the choice of artificial protein or nucleic acid scaffolds for the spatial modular organization of artificial systems (see featured research topic review by Pröschel et al. and references cited therein).

This research topic focuses on the importance of computational modeling for the rational design of synthetic systems on all these levels. Consequently, a wide variety of approaches is being covered in this issue, from pathway engineering in eukaryotic cells to molecular dynamics simulations of transport processes. The modeling approaches range from partial differential equations, allowing predictions of spatiotemporal concentration of metabolites in a biochemical microreactor (see featured research topic original research article by Elbinger et al.), to steady-state equations, testing the effect of metabolic engineering strategy in large-scale metabolic networks (see, e.g., the featured research topic article by Basler et al.). A new metabolic engineering strategy for the glucosinolate pathway that increases dihomomethionine levels without increasing the levels of leucine-derived side products was experimentally validated in *Nicotiana benthamiana* (see featured research topic original research article by Crocoll et al. and references to previous attempts therein). Sharing of modeling results and ensuring reproducibility of model-data integration requires setting and following a set of standards, which are currently established in the context of metabolomics research (see featured research topic review by Hill et al.).

The majority of the articles covered in this research topic article is contributed by members of the interdisciplinary project SynBio that is funded by the emerging fields initiative of the Friedrich-Alexander-Universität Erlangen-Nürnberg (Germany).

## Author Contributions

LV and ZN have conceived and written the article.

## Conflict of Interest Statement

The authors declare that the research was conducted in the absence of any commercial or financial relationships that could be construed as a potential conflict of interest.
